# Surgical Management of Dentigerous Cyst: A Silent Pathology With Destructive Potential

**DOI:** 10.7759/cureus.102721

**Published:** 2026-01-31

**Authors:** Effie Edsor, Prabhusankar K, Nandagopan S, Samuel Sugantharaj L, Sathya Priya B

**Affiliations:** 1 Oral and Maxillofacial Surgery, Bharath Institute of Higher Education and Research, Chennai, IND; 2 Oral and Maxillofacial Surgery, RVS Dental College and Hospital, Coimbatore, IND; 3 Oral and Maxillofacial Surgery, Sree Mookambika Institute of Dental Sciences, Kulasekharam, IND; 4 Anatomy, Sree Balaji Dental College and Hospital, Chennai, IND

**Keywords:** bone destruction, dentigerous cyst, enucleation, impacted tooth, odontogenic cyst

## Abstract

Dentigerous cysts are common odontogenic cysts associated with the crowns of unerupted teeth and are often detected incidentally due to their asymptomatic nature. This case series presents three patients with radiolucent lesions associated with impacted teeth, all of which were clinically silent yet demonstrated significant cystic expansion on radiographic examination. Surgical enucleation of the cysts along with the extraction of the associated impacted teeth was performed in all cases, and histopathological analysis confirmed the diagnosis of dentigerous cyst. Postoperative follow-up revealed satisfactory bone healing with no evidence of recurrence. These cases highlight the potential for dentigerous cysts to grow silently and cause considerable bone destruction, underscoring the importance of routine radiographic evaluation, early diagnosis, and timely surgical management to prevent complications and ensure favorable outcomes.

## Introduction

About 25% of all odontogenic cysts of the jaws are dentigerous cysts, which are the most prevalent developmental odontogenic cysts [1]. Its etiology is generally attributed to the accumulation of fluid between the reduced enamel epithelium and the enamel surface, in which the pressure exerted by the erupting tooth on the follicle impedes venous outflow and leads to the transudation of serum during its development. Over time, this process causes separation of the follicle from the crown and subsequent cyst formation. Since most of these cysts are asymptomatic and most often associated with impacted mandibular third molars and permanent maxillary and sometimes mandibular canines, they are often observed as an accidental finding on radiograph. A distinct radiolucent mass encircling the crown of an impacted tooth is the initial sign of a dentigerous cyst. Multiple tooth-encompassing dentigerous cysts are rare [2]. Sometimes, a dentigerous cyst can arise from an inflammatory etiology of a deciduous tooth [3]. Early diagnosis and appropriate surgical management are essential to prevent such complications and to preserve adjacent vital structures [4]. A dentigerous cyst can also arise due to a guided eruption of an impacted tooth [5]. This case series represents the management of a dentigerous cyst associated with impacted mandibular canines and mandibular third molar successfully treated through enucleation and also highlights the importance of clinical and radiographic evaluation and meticulous surgical technique involved in managing such lesions.

## Case presentation

Case 1

A 42-year-old man came with the complaint of pain in his lower left front tooth region for the past one month. An orthopantomogram (OPG) revealed impacted mandibular left third molar (38) and left canine (33). A well-defined unilocular large radiolucency with the impacted 33 was observed. A cone beam computed tomography (CBCT) showed a well-defined hypodense area evident in the mandibular anterior region with an irregular border, loss of trabecular pattern, and a homogenous internal structure. The impacted 33 was oriented in a horizontal direction. Superoinferiorly, the lesion extended from 4.3 mm from the alveolar crest in relation to the mandibular anteriors and 4.1 mm from the inferior border of the mandible. Mediolaterally, the lesion extended from the orifice of the mental foramen up to the distal surface of tooth 42. Mild root resorption was evident in the root apices of 44, 43, 42, 41, 31, and 32. Teeth 31, 32, 41, 42, 43, and 44 were non-vital. So the endodontic treatment was advised after that enucleation with the surgical removal of the impacted canine.

**Figure 1:**
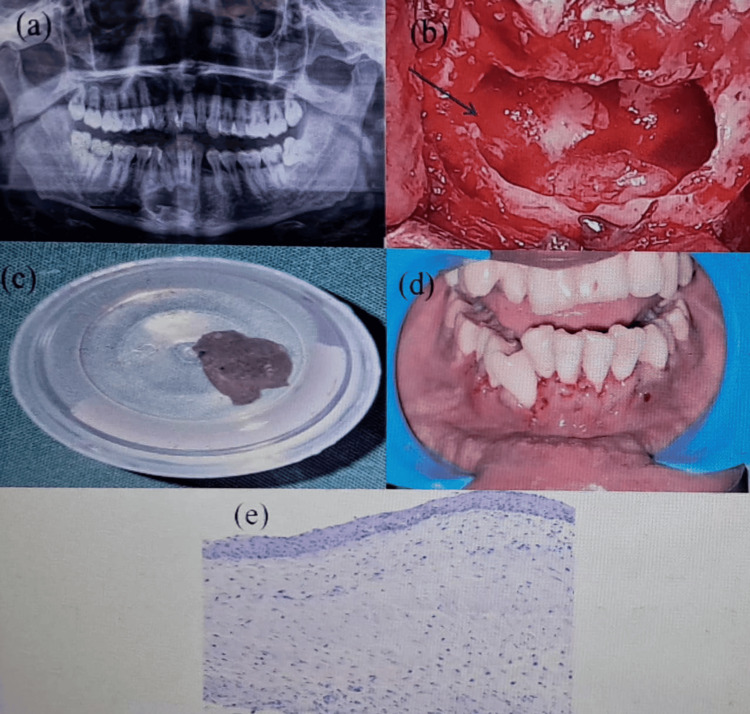
Case 1: (a) OPG revealing a well-defined unilocular radiolucency with impacted 33 (black arrow). (b) Empty cystic cavity after enucleation followed by the surgical extraction of 33 (black arrow). (c) Cystic content sent for histopathological examination. (d) Postoperative one week clinical picture showing uneventful healing. (e) Histopathological examination confirming a dentigerous cyst OPG: orthopantomogram

Under strict aseptic precautions, the patient was prepared and draped, and local anesthesia was achieved using 2% lignocaine with 1:80,000 adrenaline through bilateral mental nerve and local infiltrations. A crevicular incision was made from the left mandibular canine (33) to the right first molar (46), with vertical releasing incisions at both ends. A full-thickness mucoperiosteal flap was reflected to expose the underlying cortical bone. Thinning and expansion of the labial cortical plate were noted. A bony window was created using a round bur under copious saline irrigation, the cyst lining was carefully separated from the surrounding bone and completely enucleated, and the associated impacted mandibular canine was surgically removed. The cystic content was sent for histopathological examination, which was later confirmed as a dentigerous cyst. The cavity was irrigated with povidone-iodine and saline solution. Hemostasis was achieved, and the flap was repositioned and sutured with 3-0 black silk interrupted sutures (Figure 1).

Case 2

A 33-year-old woman came with the complaint of pain in his lower front tooth region for the past two months. OPG revealed a well-defined unilocular large radiolucency with the impacted mandibular left canine along with an irregular border with loss of trabecular pattern and homogenous internal structure. The appearance was suggestive of a single dentigerous cyst with the impacted 33 in a vertical direction. There was no evidence of root resorption; however, teeth 31, 32, 34, 35, 36, 41, 42, 43, 44, 45, and 46 were found to be non-vital. So the endodontic treatment was advised after that enucleation with the surgical removal of the impacted canine.

**Figure 2:**
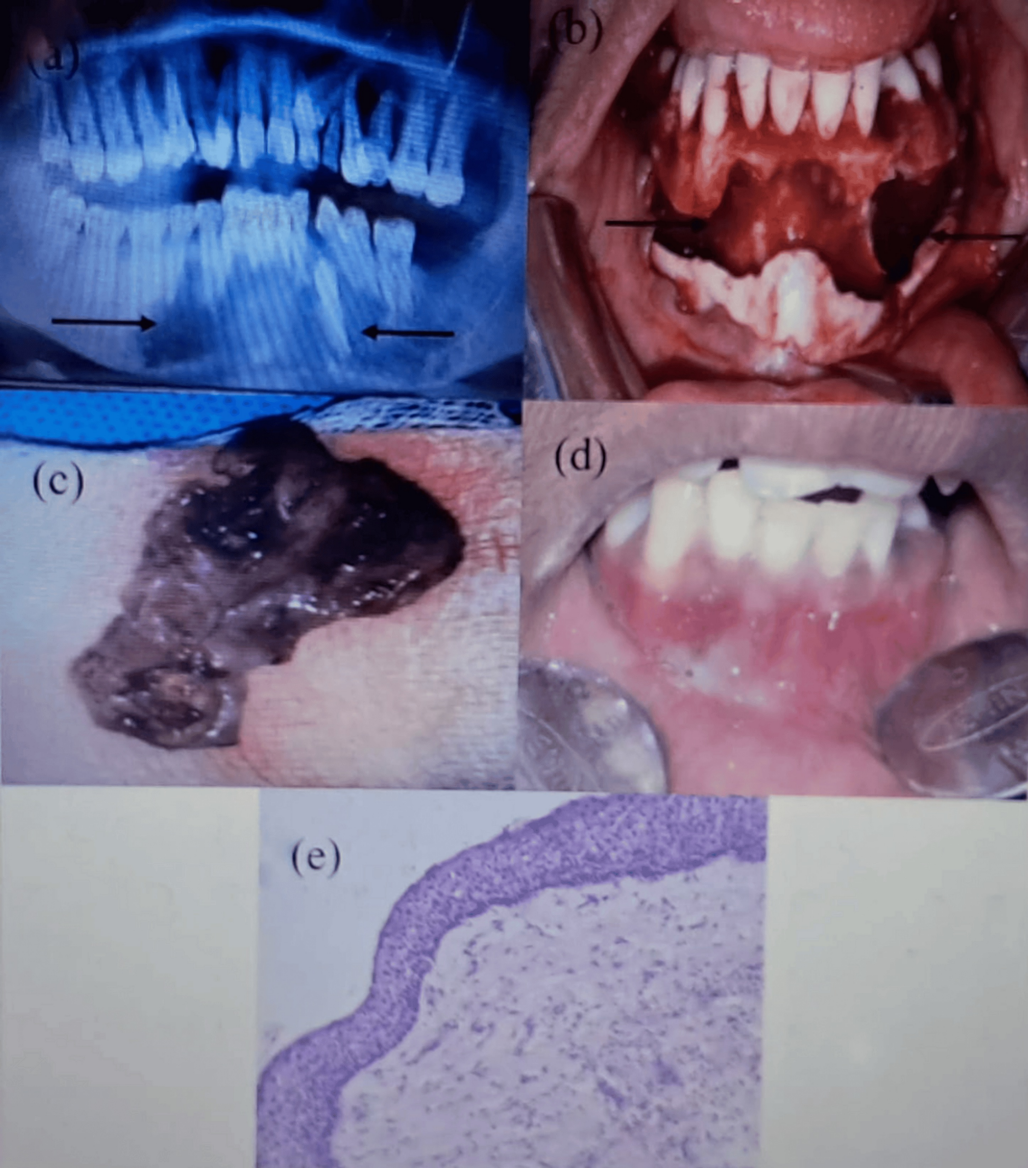
Case 2: (a) OPG revealing a large well-defined unilocular radiolucency extending from the mesial aspect of 36 to the mesial aspect of 46 with impacted 33 (black arrows). (b) Empty cystic cavity after enucleation with the surgical extraction of 33 (black arrows). (c) Cystic content which was sent for histopathological examination. (d) Postoperative one week picture showing uneventful healing. (e) Histopathological examination confirming a dentigerous cyst OPG: orthopantomogram

Under standard aseptic precautions, the patient was prepared and draped, and local anesthesia was achieved using 2% lignocaine with 1:80,000 adrenaline through the bilateral mental nerve and local infiltration block. A crevicular incision was made from the left mandibular second molar (37) to the right second molar (47), with vertical releasing incisions at both ends. A full-thickness mucoperiosteal flap was reflected to expose the underlying cortical bone. Thinning and expansion of the labial cortical plate were noted. A bony window was created using a round bur under copious saline irrigation, the cyst lining was carefully separated from the surrounding bone and completely enucleated, and the associated impacted mandibular canine was surgically removed. The cystic content was sent for histopathological examination, which later revealed a dentigerous cyst. The cavity was irrigated with povidone-iodine and saline solution. Hemostasis was achieved, and the flap was repositioned and sutured with 3-0 black silk interrupted sutures (Figure 2).

Case 3

A 28-year-old man came with the complaint of pain in his lower right front tooth region for the past two months. OPG revealed a well-defined unilocular large radiolucency with the impacted mandibular right third molar. The lesion had an irregular border with loss of trabecular pattern and a homogenous internal structure. The impacted 48 was oriented in a horizontal direction.

**Figure 3:**
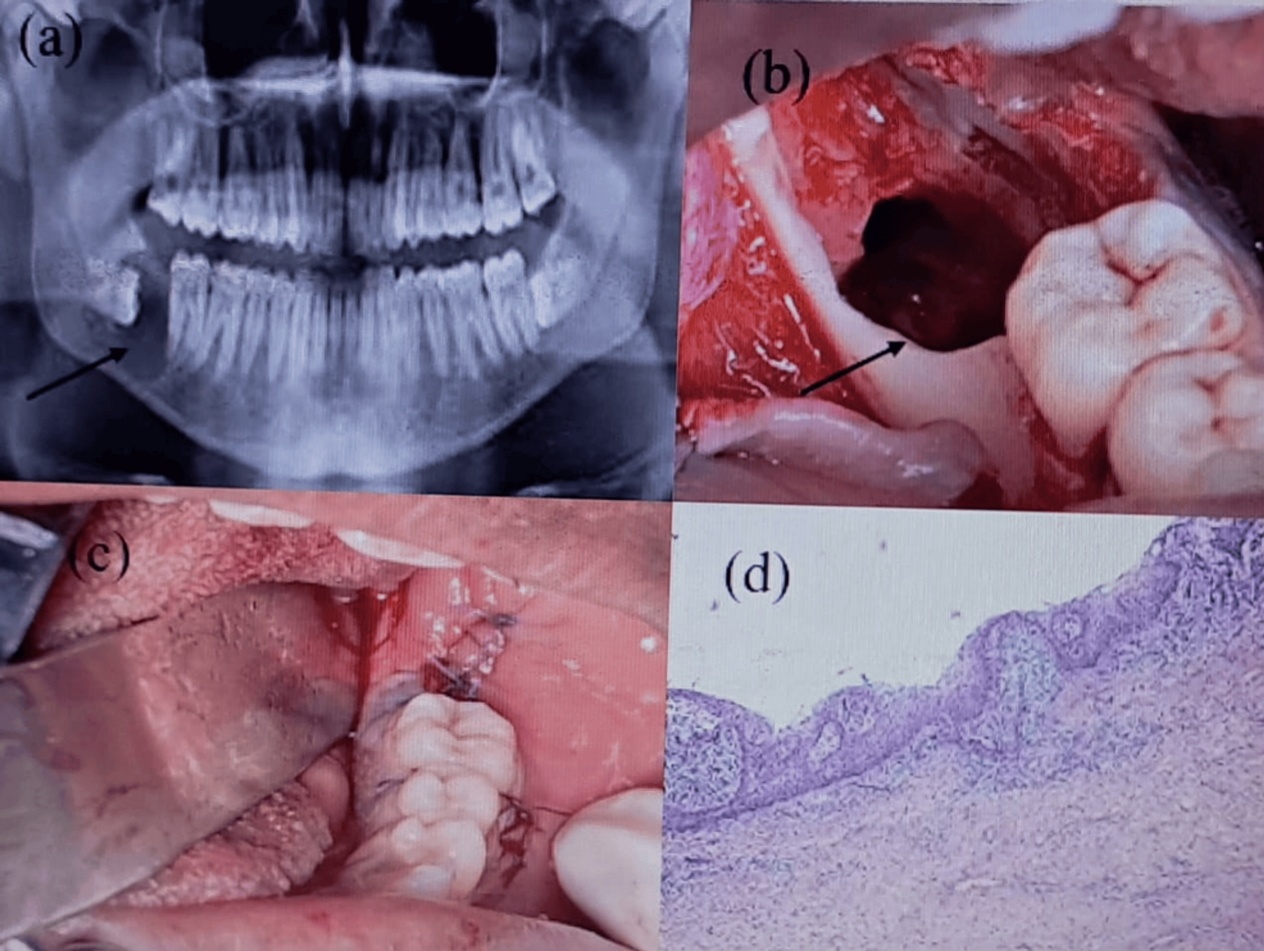
Case 3: (a) OPG revealing a well-defined unilocular radiolucency with impacted 48 (black arrow). (b) Empty cystic cavity after enucleation with the surgical extraction of 48 (black arrow). (c) Clinical picture showing immediate suturing. (d) Histopathological examination confirming a dentigerous cyst OPG: orthopantomogram

Under standard aseptic precautions, the patient was prepared and draped, and local anesthesia was achieved using 2% lignocaine with 1:80,000 adrenaline through inferior alveolar and long buccal nerve blocks. A crevicular incision was placed from 46 to the retromolar fossa region with a distal relieving incision. A full-thickness mucoperiosteal flap was reflected to expose the underlying cortical bone. The cyst lining was separated from the surrounding bone and completely enucleated. The impacted mandibular third molar (48) was surgically removed. The cystic sac was sent for histopathological examination, which later revealed a dentigerous cyst. The cavity was irrigated with povidone-iodine and saline solution, and hemostasis was achieved. The flap was repositioned and sutured with 3-0 Vicryl interrupted sutures (Figure 3).

## Discussion

Dentigerous cysts are the second most common odontogenic cysts and are typically associated with the crowns of unerupted or impacted teeth, most frequently the mandibular third molars and maxillary canines [6].

Radiographically, dentigerous cysts commonly present as well-defined unilocular radiolucencies attached at the cemento-enamel junction of an unerupted tooth [6]. In most cases, a dentigerous cyst can show a diverse radiological presentation, highlighting its diagnostic challenges [7]. However, overlap with other cystic and neoplastic lesions, such as keratocystic odontogenic tumors, unicystic ameloblastomas, or radicular cysts, requires careful differential diagnosis [8]. Radiographically, in contrast, radicular cysts are periapical lesions associated with a non-vital tooth and do not involve the cemento-enamel junction or surround the crown. Odontogenic keratocysts, although often unilocular, typically exhibit a more scalloped, anteroposterior expansion with minimal buccolingual expansion. An enlarged dental follicle can be associated with dentigerous cysts, having a follicular space of >5 mm.

Aspiration findings may aid in differentiating dentigerous cysts from other odontogenic cysts. Dentigerous cysts yield a straw-colored, pale yellow, serous fluid, reflecting their non-keratinizing epithelial lining and the transudative process responsible for cyst enlargement. In contrast, odontogenic keratocysts produce a thick, cheesy, keratin-laden aspirate, which is pathognomonic and helps distinguish them from dentigerous cysts. Radicular cysts, being inflammatory in origin, often yield turbid, brownish, or straw-colored fluid containing inflammatory exudate, and the aspirate may contain cholesterol clefts or cellular debris. Ameloblastomas typically produce no fluid or only scant hemorrhagic aspirate, supporting their solid or partially cystic nature. 

Reported transformations include ameloblastoma, squamous cell carcinoma, and mucoepidermoid carcinoma arising within longstanding cysts [9]. Piattelli et al. studied the gene Ki-67 expression in dentigerous cysts, unicystic ameloblastomas, and ameloblastomas arising from dental cysts [10]. While none of the present cases demonstrated dysplastic or neoplastic changes, these possibilities highlight the need for thorough examination of cystic lining tissues [11].

The destructive potential of dentigerous cysts is primarily related to their capacity for bone resorption and displacement of adjacent teeth [12]. Larger cysts may even lead to cortical plate thinning or perforation, which can complicate surgical management [13]. Treatment typically involves the enucleation of the cyst along with the removal of the associated tooth. In cases involving large cystic cavities, marsupialization may be considered to reduce lesion size prior to definitive surgery [14]. Most commonly, the envelope flap has been widely used due to its simplicity and broad exposure in the surgical field. However, the increased marginal incision length may increase the risk of postoperative gingival recession, particularly in cases involving the anterior maxilla or thin biotype gingiva. Secondly, the triangular flap demonstrated advantages in terms of improved surgical access and reduced tension during closure. Consistent with earlier literature, this flap allowed effective retraction and enhanced visibility in cases involving deeply seated cysts or those extending into the mandibular ramus. Nonetheless, the vertical releasing incision may predispose patients to localized scarring or transient paresthesia if placed too close to neurovascular structures. Thirdly, the semilunar flap provides a minimally invasive alternative, particularly for smaller cysts or those requiring only limited exposure [15]. The reduced involvement of marginal gingiva and the preservation of papillae are notable benefits. However, limited access and restricted visualization remain significant drawbacks, making these designs less suitable for large or complex cysts. For osteotomy, a round carbide bur no. 6 was typically used under copious irrigation to gently remove the thin cortical bone overlying the crown or to enlarge the bony window. 

## Conclusions

This case series emphasizes that dentigerous cysts, despite their silent presentation, can exhibit considerable destructive potential. Early radiographic detection and prompt surgical intervention are crucial to prevent extensive bone loss and related complications. All these cases demonstrated favorable healing with no recurrence and no nerve paresthesia, underscoring the effectiveness of timely diagnosis and appropriate management in ensuring optimal patient outcomes.
